# Disruption of erythroid nuclear opening and histone release in myelodysplastic syndromes

**DOI:** 10.1002/cam4.1969

**Published:** 2019-01-30

**Authors:** Baobing Zhao, Hui Liu, Yang Mei, Yijie Liu, Xu Han, Jing Yang, Amittha Wickrema, Peng Ji

**Affiliations:** ^1^ Department of Pathology, Feinberg School of Medicine Northwestern University Chicago Illinois; ^2^ Key Laboratory of Chemical Biology, School of Pharmaceutical Sciences Shandong University Jinan China; ^3^ Section of Hematology/Oncology, Department of Medicine The University of Chicago Chicago Illinois

**Keywords:** chromatin condensation, enucleation, erythropoiesis, myelodysplastic syndromes

## Abstract

Mammalian terminal erythropoiesis involves several characteristic phenomena including chromatin condensation and enucleation. One of the newly identified features of terminal erythropoiesis in mouse is a dynamic nuclear opening and histone release process, which is required for chromatin condensation. However, it is unclear whether the same feature is present in human. Here, we use an in vitro human CD34‐positive hematopoietic stem and progenitor cell culture system and reveal that nuclear openings and histone release are also identified during human terminal erythropoiesis. In contrast to mouse in which each erythroblast contains a single opening, multiple nuclear openings are present in human erythroblast, particularly during the late‐stage differentiation. The nuclear opening and histone release process is mediated by caspase‐3. Inhibition of caspase‐3 blocks nuclear opening, histone release, chromatin condensation, and terminal differentiation. We confirm the finding of histone cytosolic release in paraffin‐embedded human bone marrow in vivo. Importantly, we find that patients with myelodysplastic syndrome (MDS) exhibit significant defects in histone release in the dysplastic erythroblasts. Our results reveal developmentally conserved nuclear envelop and histone dynamic changes in human terminal erythropoiesis and indicate that disruption of the histone release process plays a critical role in the pathogenesis of dyserythropoiesis in MDS.

## 1 INTRODUCTION

Terminal differentiation of erythroid progenitors into mature erythrocytes is a complex and highly regulated process.^1^, ^2^, ^3^ One of the hallmark features of mammalian terminal erythropoiesis is enucleation, which requires nuclear and chromatin condensation.^1^, ^4^, ^5^ We previously revealed that histones are partially released from a nuclear opening during mouse terminal erythropoiesis.^6^ Caspase‐3 is required for nuclear opening and chromatin condensation. Inhibition or knockdown of caspase‐3 blocks nuclear opening formation, histone release, chromatin condensation, and final enucleation, leading to defects in erythroid terminal differentiation and cell death.^6^, ^7^ However, it is unclear whether the same phenomenon occurs in human terminal erythropoiesis, although caspase‐3 is known to be involved in human erythropoiesis.^8^, ^9^, ^10^, ^11^ Here, we demonstrate that caspase‐3‐mediated nuclear opening formation and histone release are present during human terminal erythropoiesis. In contrast to mouse in which each erythroblast has a single opening, there are multiple nuclear openings on a human erythroblast, especially during the late‐stage differentiation. We also found that the histone release process is disrupted in patients with myelodysplastic syndromes with dyserythropoiesis, which indicates the pathophysiological significance of nuclear opening and histone release in red cell‐related diseases.

## 2 METHODS

### 2.1 Cell Isolation and culture

Human primary erythroid progenitor cells were derived by in vitro culture of CD34+ cells isolated from growth factor‐mobilized peripheral blood. The purified CD34+ cells were cultured in a serum‐containing medium consisting of Iscoveʼs modified Dulbeccoʼs medium (IMDM) with 15% human serum, 15% fetal‐bovine serum, 1% penicillin/streptomycin, recombinant human interleukin‐3 (10 ng/mL), stem‐cell factor (SCF, 50 ng/mL), and erythropoietin (Epo, 2 units/mL) up to day 3 of culture. Subsequent cultures did not contain IL‐3 but included Epo and decreasing concentrations of SCF as described previously to promote terminal differentiation.^12^, ^13^

### 2.2 Immunofluorescence microscopy

Cells were washed in phosphate‐buffered saline (PBS), fixed in 4% paraformaldehyde for 15 minutes and permeabilized by 0.1% Triton X‐100 in PBS for 10 minutes at room temperature. Fixed cells were washed with PBS and incubated with antibodies for 1 hour. After washing three times using PBS, cells were incubated in fluorescence‐conjugated secondary antibody for 1 hour. Finally, the cells were stained with 4’, 6‐diamidino‐2‐phenylindole (DAPI). Stained cells were then mounted over a glass slide with Slowfade antifade reagent (Invitrogen) and visualized using a fluorescence microscope.

### 2.3 Statistical analyses

Statistical analysis was performed using GraphPad Prism (GraphPad Software, La Jolla, CA, USA). All data are expressed as mean ±SD except where indicated otherwise. Statistical comparisons were made using paired Student's *t *test. Statistical significance was defined as *P* < 0.05.

### 2.4 Patients and institutional review board approval

Myelodysplastic syndrome patient data were obtained following informed consent under institutional review board approved protocols at Northwestern University. The study was conducted in accordance with the Declaration of Helsinki.

## 3 RESULTS

### 3.1 Histones are partially released into the cytoplasm during human terminal erythroid differentiation

**Figure 1:**
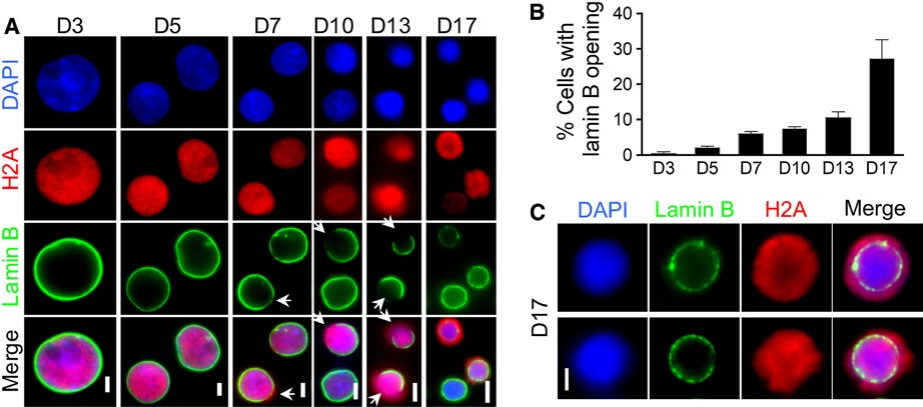
Caspase‐3‐mediated nuclear opening formation with histone release during human terminal erythroid differentiation. A, Immunofluorescent stains of lamin B and H2A in differentiating primary human erythroblasts at indicated days in culture. Arrows indicate lamin B openings. Scale bars: 5 µm. B, Quantitative analysis of the percentage of cells with lamin B opening in A. C, Immunofluorescent stains of lamin B and H2A in culture human erythroblasts on day 17. Scale bars: 2 µm

We previously demonstrated that histones are partially released from a nuclear opening during mouse terminal erythropoiesis.^6^ To determine whether the same feature is also present during human erythroid differentiation, we used an in vitro human hematopoietic stem and progenitor commitment and differentiation system, in which primary human CD34+ cells are cultured in the presence of erythropoietin and differentiate into reticulocytes.^12^, ^13^ This in vitro culture system allows cells to commit to the erythroid lineage by day 3, and go through every major stage in the erythroid differentiation in the next 14 days.^14^ We first performed immunofluorescence stains using antibodies against H2A and lamin B at differentiation stages on days 3, 5, 7, 10, 13, and 17. Early stage cells (day 3 and day 5 when the cells are in the proerythroblast stage) exhibit H2A nuclear localization with smooth and uniform lamin B stains. In contrast, lamin B gaps with lower staining intensity are observed in cells on day 7, and these gaps become larger over time. The release of histone H2A into the cytoplasm through lamin B gaps is also detected starting on day 7 (Figure 1A). The frequency of lamin B opening in erythroblasts is gradually increased during terminal differentiation (Figure 1B). However, in contrast to mouse in which one single nuclear opening is present in an erythroblast,^6^ there are multiple nuclear openings on the orthochromatic erythroblast (day 17) in human (Figure 1C). H2A is released from these openings forming a perinuclear staining pattern in most cells at this stage. Overall, these data reveal that nuclear opening and histone release occur during human terminal erythropoiesis.

**Figure 2:**
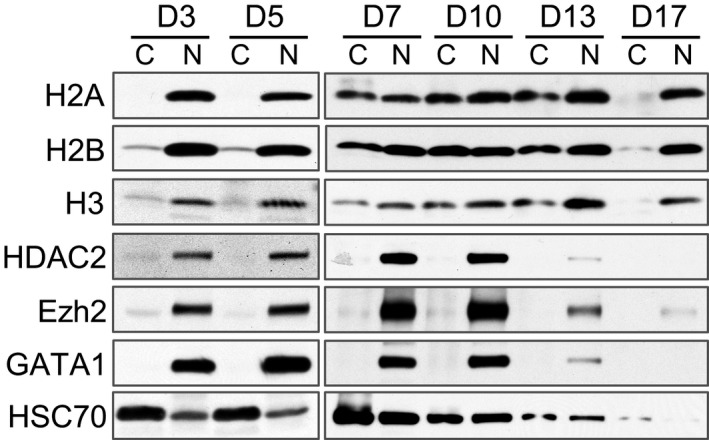
Histones are partially released into the cytoplasm during human terminal erythroid differentiation. Western blot analysis of indicated proteins from fractionated cytoplasmic (C) and nuclear (N) lysates of differentiating primary human erythroblasts on indicated days in culture with Epo. An equal number of cells were loaded in each lane

To further confirm histone release, we performed a cell fractionation experiment on human erythroblasts from different days in culture. Western blot analysis shows H2A in the cytosol fractions in cells after 7 days in culture. Cytosolic H2B and H3 are detectable at an earlier time point at day 3 and day 5 (Figure 2). Non‐histone nuclear proteins, such as HDAC2, Ezh2 and GATA1, remain in the nucleus throughout erythroid cells differentiation. These results are consistent with our previous findings in mouse.^6^

### 3.2 Caspase‐3 activation is required for nuclear condensation and histone release in human terminal erythropoiesis

**Figure 3:**
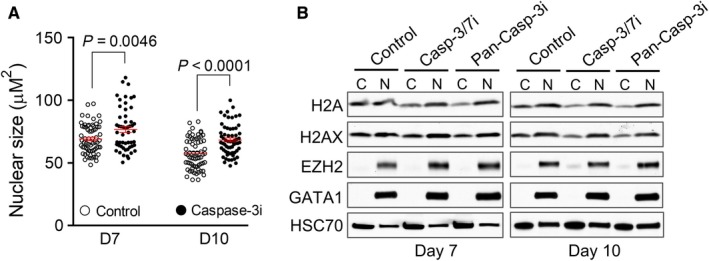
Caspase‐3 activation is required for nuclear condensation and histone release in human terminal erythropoiesis. A, The effect of caspase‐3 inhibitor on nuclear condensation. CD34+ cultured in Epo‐containing medium were treated with caspase‐3 inhibitor (Caspase‐3i) on day 3 in culture and collected on day 7 or day 10. Nuclear size was measured by calculating the DAPI‐positive area using the ImageJ software. Each dot represents a single cell. Data were obtained from three independent experiments. B, Western blot analysis of indicated proteins from fractionated cytoplasmic (C) and nuclear (N) lysates of indicated cells. CD34+ cells were treated with caspase‐3/7 inhibitor (casp‐3/7i) or pan‐caspase‐3 inhibitor (pan‐casp‐3i) on day 3 in culture and collected on day 7 or day 10. An equal number of cells were loaded in each lane

We previously revealed that in mouse erythroblasts, caspase‐3 is critical for the cleavage of lamin B and formation of nuclear opening.^6^ To determine whether caspase‐3 is also required for nuclear opening and histone release in human terminal erythropoiesis, we treated the cells with caspase‐3 inhibitors on day 3 (erythroid lineage committed).^13^, ^14^ As expected, the nuclear size is significantly increased in the present of a caspase‐3 inhibitor, suggesting that chromatin and nuclear condensation is blocked by caspase‐3 inhibition (Figure 3A). Compared to the control cells, histone release is significantly inhibited in cells treated with caspase‐3 inhibitors (Figure 3B).

**Figure 4:**
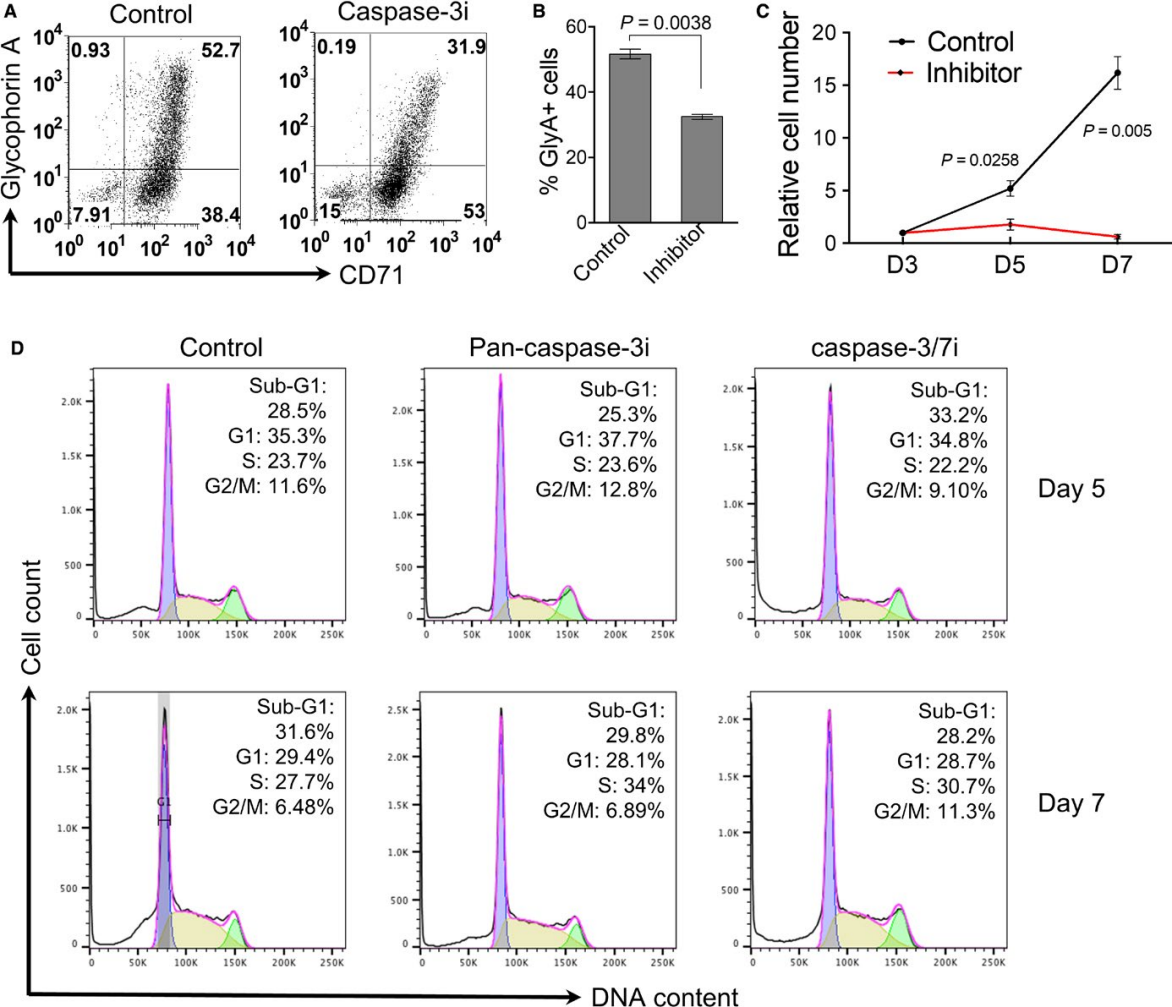
Caspase‐3 inhibition leads to the blockage of human erythroid cells differentiation and proliferation. A, CD34+ cells cultured in Epo‐containing medium were treated with caspase‐3 inhibitor on day 3 in culture and collected on day 7. Flow cytometric analysis of transferrin receptor (CD71) and glycophorin A were performed. B, Quantitative analysis of the percentage of glycophorin A positive cells (GlyA+) in A. Data were obtained from three independent experiments. C, CD34+ cells cultured in Epo‐containing medium were treated with caspase‐3 inhibitor on day 3, and the total cell number was counted on indicated days in culture. Data were shown as fold changes (day 3 as 1) and obtained from three independent experiments. D, Flow cytometric analysis of cell cycle distribution. Cells were treated as in C and analyzed by flow cytometry using propidium iodide (PI)‐staining. The percentages of populations of different cell cycle phases are shown in the upper‐right corner. The data are representative of three independent experiments

Furthermore, flow cytometric analysis reveals that caspase‐3 inhibitor treatment leads to an inhibition of erythroid cells differentiation in which the CD71 and glycophorin A double positive cells are significantly decreased (Figure 4A,B). In addition, caspase‐3 inhibitor also significantly blocks cell proliferation (Figure 4C). There are no detectable effects on cell cycle progression (Figure 4D).

### 3.3 MDS patients exhibit reduced nuclear opening formation and histone release in dysplastic erythroblasts

**Figure 5:**
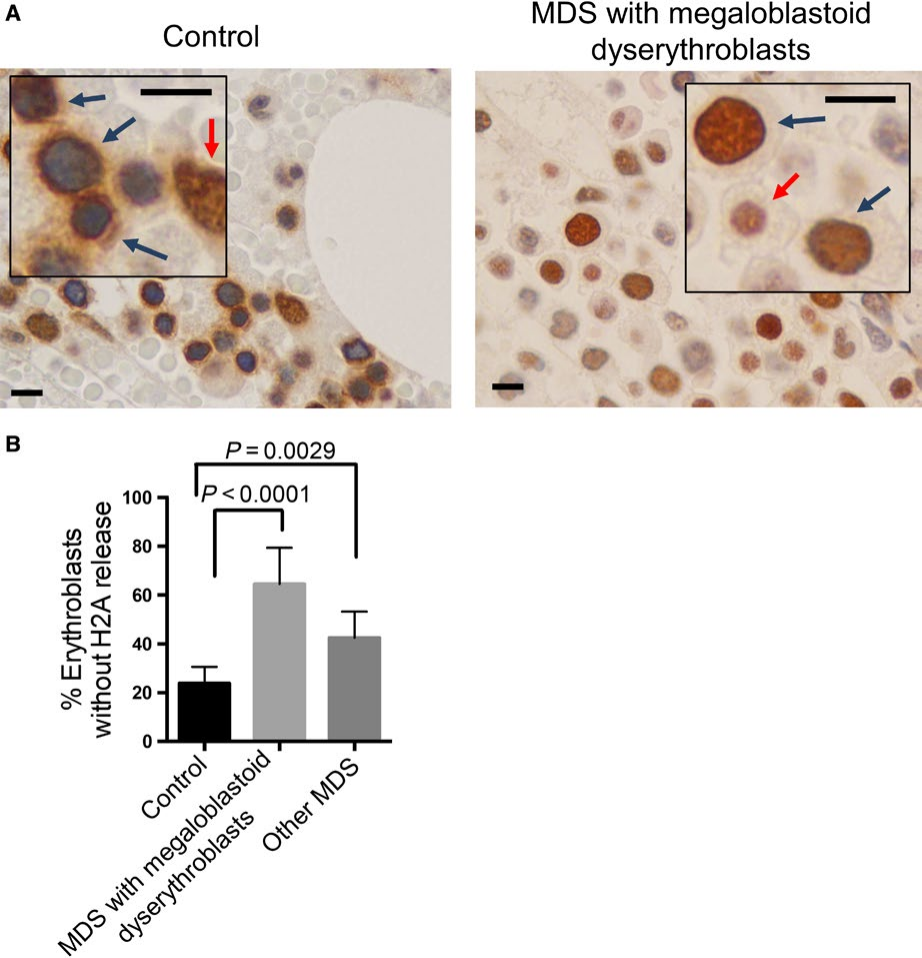
Myelodysplastic syndrome (MDS) patients exhibit reduced nuclear opening formation and histone release in erythroblasts. A, Representative immunohistochemical stains of H2A in normal control individuals and patients with myelodysplastic syndromes carrying megaloblastoid erythroblasts. Blue arrows indicate erythroblasts. Red arrows indicate other hematopoietic cells. Scale bars: 8 μm. The unstained cells are red blood cells due to hemorrhage during biopsy. B, Quantitative analysis of the percentage of erythroblasts without H2A release. Control: N = 6, MDS with megaloblastoid erythroblasts: N = 10, Other MDS: N = 7. 500 erythroblasts were counted in each case

Defects in chromatin condensation in the erythroid lineage are commonly seen in patients with megaloblastic anemia and myelodysplastic syndromes (MDS).^1^, ^4^, ^15^, ^16^ The pathogenesis of these defects is not clear. Given the significance of nuclear opening with histones release in chromatin condensation and human erythroid differentiation, we investigated the dynamics of histone distribution in bone marrow erythroblasts from MDS patients with megaloblastoid erythroblasts and normal healthy donor controls. In human adult bone marrow, orthochromatic erythroblasts comprise a large proportion of the erythroid cells that are easily identifiable by their round and condensed nuclei.^14^ Consistent with the immunofluorescent results, immunohistochemical stain reveals a perinuclear and cytoplasmic localization pattern of H2A in the orthochromatic erythroblasts from healthy donors. In contrast, cells of other lineages show regular nuclear staining pattern (Figure 5A left). When we examine the MDS cases with megaloblastoid changes of dyserythropoiesis, H2A is nearly absent from the cytoplasm but shows a strong nuclear stain in most erythroblasts (Figure 5A right). Statistical analysis from a group of MDS patients with megaloblastoid dyserythropoiesis shows markedly increased number of erythroblasts lacking H2A release. Erythroblasts from other MDS patients (no clear megaloblastoid changes of dyserythropoiesis) also exhibit mild defects in cytosolic distribution, but is less severe (Figure 5B).

## 4 DISCUSSION

Global nuclear and chromatin condensation are unique features in mammalian terminal erythropoiesis, which is essential for enucleation.^4^, ^5^ Our study demonstrates that caspase‐3‐mediated nuclear opening formation and histone release are also present during human terminal erythropoiesis, which is similar to mouse terminal erythropoiesis. This evolutionally conserved process is clearly necessary for mammalian erythroid chromatin condensation. However, it is unclear whether nuclear opening and histone release are required for enucleation in mammals, since chromatin condensation, but not enucleation, occurs during erythropoiesis in other species. Future cross‐species comparison would provide clues on this important question in red blood cell development.

Erythroid dysplasia is one of the hallmark features of MDS. Dyserythropoiesis is found in more than 80% of early‐stage MDS.^15^ Nuclear changes like abnormal chromatin clumping, bi/multinuclearity or nuclear bridges are features of erythroid cell dysplasia.^17^, ^18^ Caspase‐3 inhibition leads to the blockage of histones release and nuclear condensation, and differentiation in human terminal erythropoiesis (Figures 3 and 4). Compared to healthy individuals, erythroblasts from MDS patients exhibit impaired histones release and chromatin condensation, which phenocopies the effect of caspase‐3 inhibition. These findings indicate that defects of nuclear opening formation and histones release contribute to the pathogenesis of megaloblastoid changes in dyserythropoiesis in MDS. We have shown in our mouse study that defects in nuclear opening and histone release lead to cell death.^6^ In this respect, it is well known that increased cell death is common in MDS. While most of the cell death pathways are perhaps related to the upregulation of caspases, which is indeed the case in MDS, it is possible that caspase‐independent cell death could play an important role during the development of MDS.

Nuclear lamins exhibit distinct expression patterns and different assembly properties.^19^, ^20^ The ratio of lamin A and lamin B1 directly modulates hematopoietic programs. Erythroid differentiation is promoted by high lamin A and low lamin B1 expression.^21^ Cells expressing lamin mutants fail to show signs of chromatin condensation and nuclear shrinkage typical of apoptotic cell death.^22^ Homozygous lamin B mutant mice survive embryonic development but die at birth with defects in the lungs and bone. Cells from mutant embryos grow under standard cell culture conditions but display impaired differentiation.^23^ Given the defective histone release in MDS, it is possible that the expression pattern of different lamins is altered. There are also possibilities that certain mutations are present in lamins in patients with MDS that lead to failure in nuclear opening and histone release.

In summary, our study reveals that human terminal erythropoiesis involves caspase‐3‐mediated nuclear openings and histone release. Distinct from mouse, late‐stage erythroblasts (orthochromatic erythroid cells) tend to have multiple openings where major histones release into the cytoplasm and form a perinuclear localization pattern. Disruption of the histone release process could play a critical role in the pathogenesis of dyserythropoiesis in MDS.

## CONFLICT OF INTEREST

None declared.
